# Thermal and Optical Properties of CdS Nanoparticles in Thermotropic Liquid Crystal Monomers

**DOI:** 10.3390/ma3032069

**Published:** 2010-03-19

**Authors:** Hooi Ling Lee, Issam Ahmed Mohammed, Mohammed Belmahi, Mohamed Badreddine Assouar, Hervé Rinnert, Marc Alnot

**Affiliations:** 1 Nanoscience Research Laboratory, School of Chemical Sciences, University Sains Malaysia, 11800 Penang, Malaysia; 2 School of Industrial Technology, University Sains Malaysia, 11800 Penang, Malaysia; 3 Institut Jean Lamour, UMR 7198 CNRS, Nancy Université, Université P. Verlaine Metz, Faculté des Sciences et Techniques B.P. 239 F-54506 Vandoeuvre les Nancy Cedex, France E-Mails: badreddine.assouar@lpmi.uhp-nancy.fr (M.B.A.) ; herve.rinnert@lpm.u-nancy.fr (H.R.); marc.alnot@lpm.u-nancy.fr (M.A.)

**Keywords:** chalcogenides, nanomaterials, composite materials, liquid crystals, optical properties, *in situ* methods

## Abstract

Two new mesogenic monomers, namely 3,3’-dimethoxy-4,4’-di(hydroxyhexoxy)-N-benzylidene-o-Tolidine (**Ia**) and 4,4’-di(6-hydroxyhexoxy)-N-benzylidene-o-Tolidine (**IIa**), were reacted with cadmium sulfide (CdS) via an *in situ* chemical precipitation method in ethanol to produce CdS nanocomposites. A series of different mass compositions of CdS with **Ia** and **IIa** ranging from 0.1:1.0 to 1.0:1.0 (w/w) were prepared and characterized using X-ray Diffraction (XRD), Raman spectroscopy, Fourier Transform Infrared Spectroscopy (FT-IR), Transmission Electron Microscopy (TEM), Polarizing Optical Microscopy (POM) and Differential Scanning Calorimetry (DSC), X-ray Photoelectron Spectroscopy (XPS) and Photoluminescence Spectroscopy (PL). XRD showed that the broad peaks are ascribed to the formation of cubic CdS nanoparticles in both **Ia** and **IIa**. The average particle size for both nanocomposites was less than 5 nm with a narrower size distribution when compared with pure CdS nanoparticles. The analyses from POM and DSC demonstrated that mass composition from 0.1:1.0 up to 0.5:1.0 of CdS:**Ia** nanocomposites showed their enantiotropic nematic phase. On the other hand, polarizing optical microscopy (POM) for **IIa** nanocomposites showed that the liquid crystal property vanished completely when the mass composition was at 0.2:1.0. PL emissions for CdS: **Ia** or **IIa** nanocomposites indicated deep trap defects occurred in these both samples. The PL results revealed that addition of CdS to **Ia** monomers suppressed the photoluminescence intensity of **Ia**. However, the introduction of CdS to **IIa** monomers increased the photoluminescence and was at a maximum when the mass composition was 0.3:1.0, then decreased in intensity as more CdS was added. The XPS results also showed that the stoichiometric ratios of S/Cd were close to 1.0:1.0 for both types of nanocomposites for a mass composition of 1.0:1.0 (CdS:matrix).

## 1. Introduction

CdS is a group II–VI semiconductor, and as such, CdS nanoparticles have generated great interest due to their unique size-dependent chemical and physical properties [1]. Extensive research has focused on the synthesis of various CdS nanostructures. CdS has a bandgap energy of 2.42 eV [2,3] at room temperature, and it shows great potential for uses in photochemical catalysis, solar cells, non-linear optical materials and various luminescence devices [4].

In recent years, monomer and polymer liquid crystal (LC) nanocomposites have been widely investigated. Thermotropic liquid crystal compounds and CdS nanoparticles are frequently used in photonic applications, and a hybrid of these two materials is expected to demonstrate further enhancements in the photonic properties. Several studies have investigated the synthesis of thermotropic LC polymer materials that are composed of silver nanoparticles [5,6] and CdSe [7]. Many semiconductor/organic nanocomposites (e.g., CdS/polyvinyl alcohol [8], CdS/N-polyvinylcarbazole [9] and CdS/polystyrene [10]) have been synthesized using various methods. Nevertheless, there are few reports on the incorporation of nanoparticles into liquid-crystalline materials. Several researchers have studied organoclay nanocomposites [11,12] that are based on thermotropic liquid-crystalline polymers. In most of the reported work, common polymers, such as epoxy resin, polystyrene and polyvinyl alcohols (PVA), are used as the matrix in the CdS/polymer nanocomposites. To the best of our knowledge, few researchers have reported the incorporation of CdS nanoparticles into thermotropic liquid crystal azomethine monomers, although Khiew and co-workers [13,14] synthesized PbS and CdS nanoparticles in lyotropic liquid crystals using a reverse microemulsion technique. In our study, azomethine was selected for this study because it belongs to a group of high performance materials [15] that exhibit excellent thermal stability [16], desirable mechanical properties [17] and environmental stability [18]. Hence, they have potential for optoelectronic, photonic and magnetic applications. Fundamental research has also suggested that the combination of CdS with monomer or polymer nanocomposites is particularly promising because this combination exhibits high aspect ratios and small sizes. As such, a number of efforts have focused on preparing CdS nanocomposites. The practical applications of thermotropic liquid crystal monomers or polymers are limited compared to conventional thermoplastic polymers because the thermotropic liquid crystal monomers are relatively expensive [19]. A major remaining challenge involves synthesizing these monomers or polymers so that they exhibit liquid crystal properties. In addition, CdS nanoparticles tend to agglomerate due to their high surface areas [8], and this agglomeration is a common problem faced by nanomaterials researchers.

This paper describes a scientific study that focuses on the synthesis and characterization of CdS nanocomposites with an emphasis on photoluminescence applications. Various methods were used, including solvothermal [3], reversed micelle [13], organosol [8], and thermolytic synthetic methods [10]. We recently published a paper on the synthesis of CdS nanoparticles using the chemical precipitation method [20]. This method is an easy, inexpensive, and single-step method. In addition, the preparation of nanocomposites is straightforward: it requires a simple reflux apparatus (a hot plate and a silicon oil bath) that is available in most research laboratories. In the current study, two different azomethine monomers were used to prepare the two types of nanocomposites. One of the monomers has a methoxy group at the meta position of the phenyl group that is attached to the aliphatic chain whereas the other monomer does not have a methoxy group attached to the phenyl group (see Section 3.2). Our goal was to use a similar *in situ* approach for preparing the CdS nanocomposites using CdS nanoparticles and aromatic azomethine monomers. The differences between the two resulting types of nanocomposites were then investigated comprehensively using various characterization methods. In addition, we investigated the photoluminescent properties of both the CdS nanoparticles and the nanocomposites at different mass compositions. We expect the photoluminescence of these newly synthesized nanocomposites to increase with greater concentration of CdS nanoparticles.

## 2. Results and Discussion

### 2.1. Microstructure and Purity Characterizations

**Figure 1:**
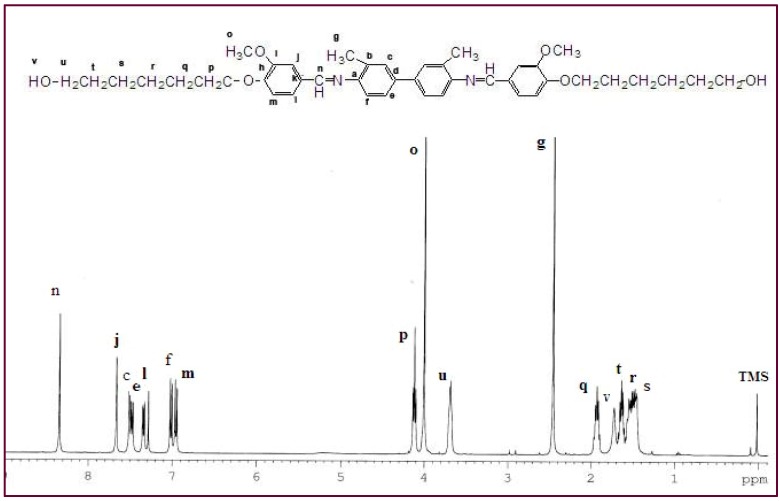
^1^H-NMR spectrum of **Ia**.

The structural characteristics of **Ia** and **IIa** were confirmed by ^1^H-NMR spectroscopy. The NMR spectrum for **Ia** (Figure 1) confirmed the presence of the azomethine group (-CH=N-) at δ = 8.33 ppm. Two characteristic singlet peaks centered at 4.03 and 2.46 ppm were due to the protons in the methoxy group (-OCH_3_) and the methyl group in o-tolidine (Ph-CH_3_), respectively. No peak was detected at δ = 4.03 ppm for **IIa**, indicating the absence of a methoxy group (-OCH_3_). A multiplet in the range of δ = 7.00–7.68 ppm was observed for both **Ia** and **IIa** and these peaks corresponded to the protons in the aromatic rings. The appearance of a multiplet at δ = 1.46–1.95 in **Ia** and **IIa** was attributed to the presence of aliphatic chains (-(CH_2_)_6_-) in these two mesogenic diols.

**Figure 2:**
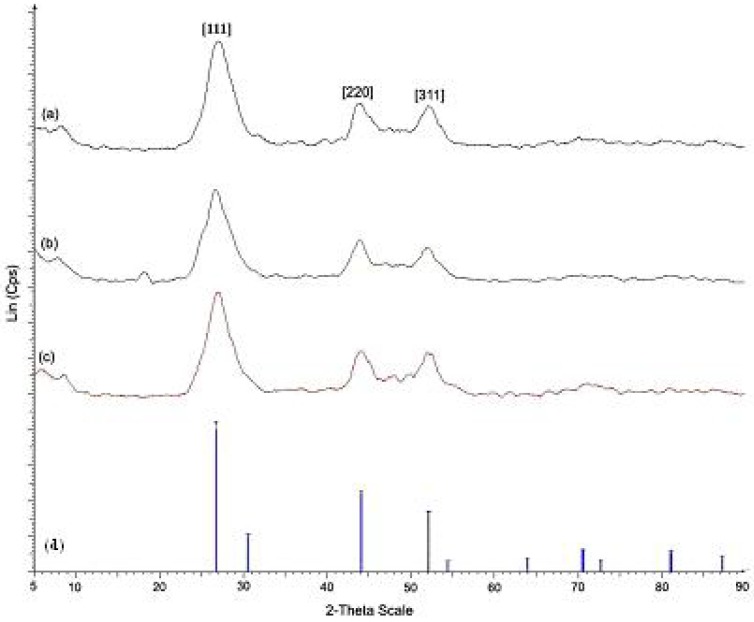
X-ray diffraction patterns of (a) CdS nanoparticles (b) CdS: **Ia** nanocomposites (1.0:1.0) (c) CdS: **IIa** nanocomposites (1.0:1.0) (d) cubic CdS (JCPDS 030-65-2887).

**Figure 3:**
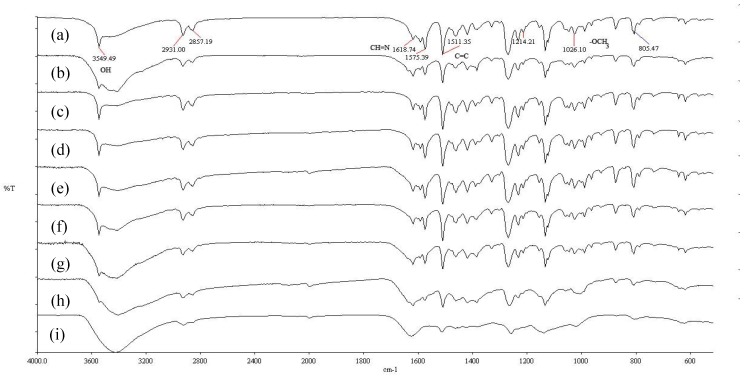
FT-IR spectra for CdS: **Ia** nanocomposites of (a) pure **Ia** and (b-i) mass compositions of 0.1:1.0 to 1.0:1.0 (w/w).

Figure 2 shows the XRD patterns for pure CdS nanoparticles and CdS nanocomposites of **Ia** and **IIa** at the mass composition of 1.0:1.0. The XRD patterns obtained for both pure CdS and CdS nanocomposites corresponded to pure cubic CdS when compared with the standard reference (JCPDS 03-065-2887). Three peaks with 2θ values of 26.6, 44.0 and 52.0 appeared in the spectrum of each sample and may be assigned to the [111], [220] and [311] Miller indices. FT-IR measurements were carried out for the CdS nanocomposites that were synthesized with different mass compositions, and these measurements were compared with the data that was obtained for pure **Ia** and **IIa**. The FT-IR analysis of pure **Ia** showed an absorption band at 1618 cm^-1^, indicating the presence of the azomethine group (-CH=N-). The peaks at 1576 cm^-1^ and 1511 cm^-1^ were due to aromatic ring skeletal vibrations and C=C bond stretching [21], respectively. The peak at 1026 cm^-1^ could be assigned to the methoxy group (-OCH_3_) present in **Ia**. The peak at 808 cm^-1^ confirmed the presence of *para*-disubstitution on the aromatic rings; this peak was associated with the bending mode of aromatic ring hydrogen atoms [21]. The peaks at 3423 cm^-1^ and 3550 cm^-1^ could be assigned to the hydroxyl groups (–OH). The spectra of CdS:**Ia** nanocomposites (Figure 3) showed a change in peak intensity in the 3423–3550 cm^-1^ range for mass compositions of 0.2:1.0 (w/w) and above, but the other peaks in the FT-IR spectra remained unchanged. Similar assignments were observed in the FT-IR spectrum for **IIa**. However, no absorption band associated with the methoxy group was found in **IIa**. The broadening of the peaks could be attributed to interactions of the polar groups (e.g., the -OH groups) in **Ia** or **IIa** with the surface of a nanoparticle via a physical mechanism such as van der Waals forces, dipole interactions or weak hydrogen bonds [7].

**Figure 4:**
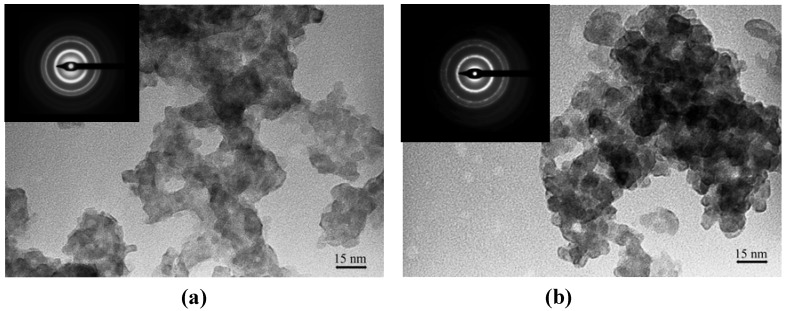
TEM images of (a) **Ia** and (b) **IIa** at mass composition of 1.0:1.0 (CdS: matrix, w/w) Insert: SAED images of CdS: matrix nanocomposites (a) **Ia** (b) **IIa** at mass composition of 1.0:1.0 (CdS: matrix, w/w).

The TEM micrographs (Figure 4) show that the particles were distinguishable, but not well-resolved due to the presence of the matrix. However, the particles were not aggregated into a big structure, although the particles were in contact with each other. Most of the particles were similar in size and have irregular rounded shapes. The average particle size for both nanocomposites was less than 5.0 nm with a standard deviation of less than 1.0 nm.

The SAED images for each nanocomposite indicated that the measurements of major *d*-spacings corresponded with the *d*-spacings obtained from Pearson’s Crystal Data database: 1120352 of CdS hexagonal crystalline phase. The three major *d*-spacings obtained for the CdS:**Ia** nanocomposite were 0.338 nm, 0.205 nm and 0.174 nm, whereas for the CdS:**IIa** nanocomposite, the major *d*-spacings were 0.343 nm, 0.210 nm and 0.177 nm. These *d*-spacings were measured from the three major rings and were assignable to the [002], [110] and [201] Miller indices. As mentioned earlier, the XRD spectrum suggested that the CdS is in the cubic crystalline phase. The observed inconsistency could be ascribed to the broadened peaks from the spectra as well as to the overlapping peaks between the XRD line of the hexagonal and cubic structures [22].

**Figure 5:**
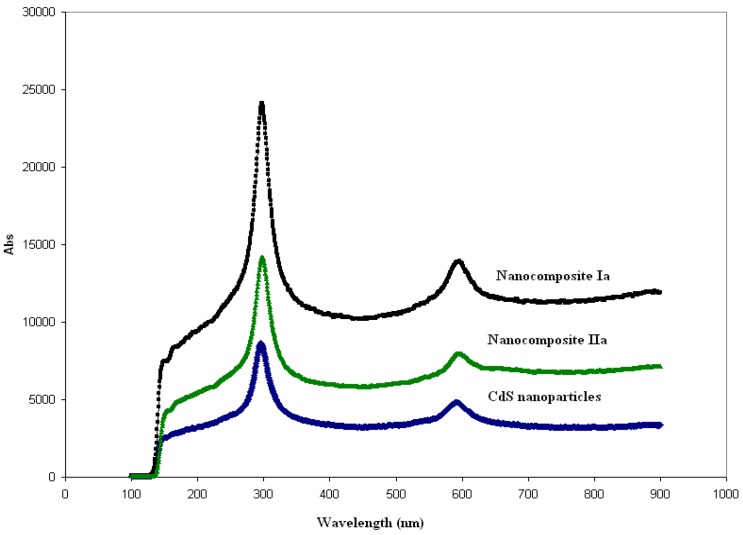
Raman spectra for pure CdS and CdS nanocomposites for **Ia** and **IIa** at the mass composition (1.0:1.0, w/w).

Raman scattering analyses were performed on pure CdS and CdS nanocomposites of **Ia** and **IIa** at 15 ºC. CdS is known to be Raman active [23] and, thus, could not be observed in the FT-IR spectra. Two characteristics of CdS LO phonon peaks were observed in all three samples (see Figure 5). The 1-LO and its much weaker overtone, 2-LO, could be observed at 292 cm^-1^ and 591 cm^-1^, respectively, in both CdS nanocomposite samples when compared with the as-prepared pure CdS. The result was in agreement with the reports by other researchers [24,25]. These researchers have observed the surface mode peak of small CdS crystallites (size = 1 μm) at 292 cm^-l^ as well as its second order peak at 596 cm^-l^. We also observed that the Raman intensity is the highest in the CdS nanocomposite of **Ia**, followed by the CdS nanocomposite of **IIa** and finally by pure CdS. The intensity of the change in the Raman bands could be influenced by the presence of **Ia** and **IIa** in the samples. When analyzed by PL spectroscopy, pure **Ia** and **IIa** have their own photoluminescence intensity values that also showed similar trends with the Raman pattern, as depicted in Figure 5.

**Figure 6:**
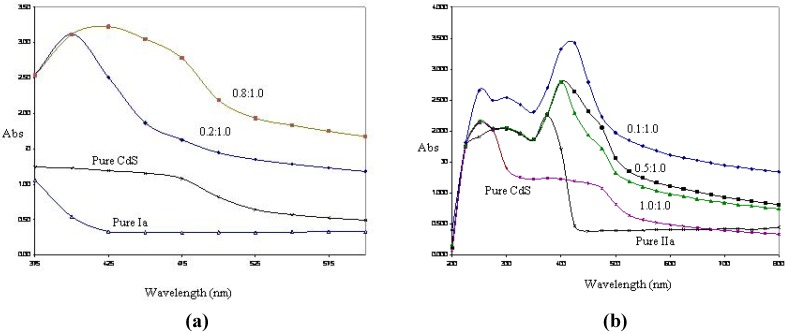
(a) UV/Vis spectroscopy analyses for pure CdS, pure **Ia** and CdS: **Ia** nanocomposites at 0.2:1.0 and 0.8:1.0 (w/w); (b) UV/Vis spectroscopic analyses for pure CdS, pure **IIa** and CdS: **IIa** nanocomposites at 0.1: 1.0, 0.5:1.0 and 1.0:1.0 (w/w).

UV/Vis spectroscopic analyses were performed on CdS:nanocomposites of **Ia** and **IIa** at selected mass composition values (Figure 6a and Figure 6b). Shoulder peaks of CdS:nanocomposite of **Ia** occurred at 450 nm and 475 nm for the mass compositions of 0.2:1.0 and 0.8:1.0. However, the absorbance band became broader as the mass composition of CdS was increased. A similar result was found with the CdS:nanocomposite of **IIa**. The absorption bands gradually broadened as the mass composition of CdS increased, and the UV absorption for **Ia** and **IIa** shifted to a shorter wavelength (blue shift) with respect to that of the bulk CdS (520 nm). This observation indicated that quantum confined electronic behavior occurred in these nanocomposites.

### 2.2. Thermal and Optical Behavior

**Figure 7:**
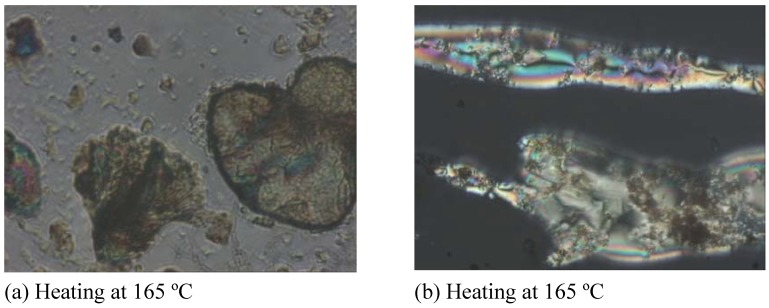
Polarizing Optical Microscope (POM) micrographs of (a) CdS: **Ia** nanocomposites and (b) CdS: **IIa** nanocomposites at 0.1:1.0 (w/w).

The phase transition temperatures of the CdS nanocomposites for **Ia** were measured using differential scanning calorimetry (DSC). The melting (T*m*) and isotropization (T*i*) points were clearly identifiable from the DSC thermograms. The mesophase of the nanocomposites was studied by polarizing optical microscopy. The mesophase was recognized from the basic textures exhibited by the mesogenic diols when viewed between the crossed polarizers in the microscope. The sample showed the Schlieren threaded texture, which confirmed the nematic phase, as shown in Figure 7.

The melting and isotropization points for pure **Ia** were 171.0 ºC and 188.7 ºC, respectively, when analyzed by DSC. Generally, the introduction of CdS nanoparticles into **Ia** does not decrease either the melting point or the isotropization point significantly. However, a slight decrease of transition temperature (Cr-N) was observed for the 0.4:1.0 and 0.5:1.0 **Ia** nanocomposites. The liquid crystal property disappeared when the mass composition of CdS:**Ia** was 0.6:1.0 or higher. No transition temperatures were observed, and only a single melting peak was detected in the DSC spectrum for the sample with a mass composition of 0.6:1.0. When the mass composition was between 0.8:1.0 and 1.0:1.0, no melting point was observed. In the case of the CdS nanocomposite of **IIa**, the melting and isotropization points for pure **IIa** are 160.1 ºC and 275.2 ºC, respectively. A different observation was noted when compared to the earlier CdS:**Ia** nanocomposite system. When 10% CdS nanoparticles (w/w) were introduced into the system, the melting point of this sample was decreased by 7 ºC to 153.1 ºC even though the isotropization temperature (273.5 ºC) did not change significantly. The melting point and the isotropization point could not be detected when the mass composition was increased to 0.2:1.0. This result was common to all samples with mass compositions between 0.3:1.0 and 1.0:1.0.

The presence of a *m*-OCH_3_ substituent on the aromatic ring reduced the clearing (isotropization) temperature from 275.2 ºC in pure **IIa** to 188.7 ºC in pure **Ia**. When substituents like a methoxy group are present at the *ortho* or *meta* positions of the central aromatic system, thermal depression and the instability of the mesophase could be observed [26,27]. Yeap and co-workers (2006)[26] suggested that the nematogenic behavior observed in some of their compounds that had a methoxy group in the *meta* position of the aromatic system could have weakened the lateral interactions between molecules, which are important for promoting higher mesophase stabilities. This effect could also be attributed to the overall anisotropic broadening of the molecule and could eventually affect the cooperative packing that is needed in the mesophase [28]. Based on the DSC analyses, **IIa** was shown to be a more stable liquid crystal because the difference between the melting point and the isotropization point was larger compared to that of **Ia.** There were slight decreases in the transition temperature of Cr-N and N-I in the **Ia** nanocomposite series. However, these decreases were relatively insignificant unless the mass concentrations were increased to 0.4:1.0 or above. When more CdS nanoparticles were added to the matrix of **Ia**, the nanoparticles functioned as a new additive material and hence, the transition temperature decreased relative to pure **Ia**. In our case, we observed two different scenarios in which the addition of CdS to **Ia** still showed the transition temperature at a discrete mass composition (0.5:1.0) and eventually lost its liquid crystalline properties. In contrast, the presence of CdS in **IIa** slightly inhibited the liquid crystal properties when compared with the **Ia** nanocomposite system. This result could be attributed to the presence of CdS nanoparticles, which effectively inhibit monomer re-crystallization depending on the concentration of CdS nanoparticles and on the type of monomers. The addition of filler (CdS nanoparticles) restricts the segmental motion of the monomers and, thus, reduces the re-crystallization process [29]. This segmental motion allows rearrangement of the monomer chains into ordered structures, thus forming crystals during the cooling process [29].

**Figure 8:**
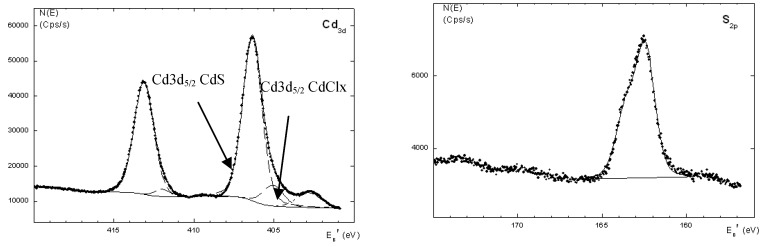
The XPS spectra of CdS: **Ia** nanocomposites.

Figure 8 shows the XPS spectra of the CdS :nanocomposite of **Ia** at a mass composition of 1.0:1.0. We detected two strong peaks of Cd3d in both samples. The binding energies for Cd3d _5/2_ and Cd3d_3/2_ were 407.5 eV and 414.4 eV, respectively, with the S2p energy at 163.6 eV. For instance, the doublet peaks that were assignable to Cd3d_5/2_ and Cd3d_3/2_ were observed at 406.4 eV and 413.3 eV (Figure 8) in the CdS: nanocomposite of **Ia** (1.0:1.0). The binding energy for S2p was 162.9 eV. The XPS results also revealed the presence of C and O, which originated from the matrix of Ia. The C1s core level was observed at a binding energy 285.1 eV and could be assigned to the hydrocarbon CH_2_ groups [30]. The presence of O1s at a binding energy of 533.2 eV could be due to H_2_O. However, there was a slight impurity of Si in the sample, which was attributed to etching of silica from the reaction flask during the acidic cleaning process.

There were two Cd3d_5/2_ peaks, namely Cd3d_5/2_ CdS and Cd3d_5/2_ CdClx. The peak for Cd3d_5/2_ CdClx occurred at around 405 eV, depending on the charge effect during the analysis. When we plotted the area under the curve of the Cd3d_5/2_ CdClx peak over the area under the curve of the Cd3d_5/2_ CdS peak for both nanocomposites, we found that the ratio slightly increased as the concentration of CdS in the nanocomposite increased. This observation could indicate that sulfur vacancies occur as the concentration of CdS increases in the samples.

**Figure 9:**
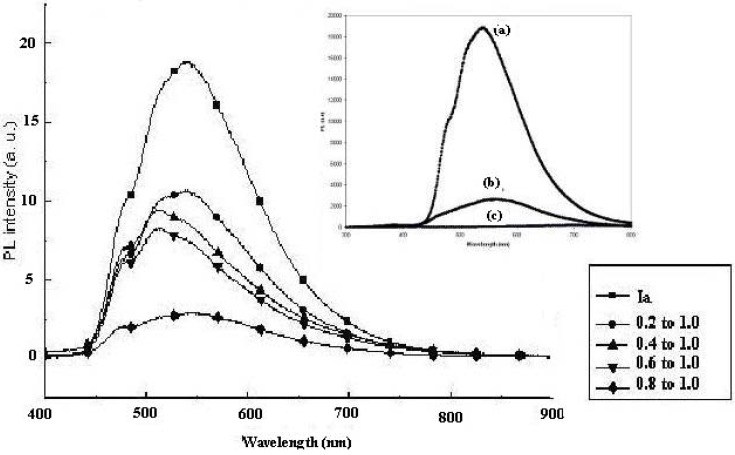
Photoluminescence study of CdS: **Ia** nanocomposites at different mass compositions (w/w); Insert: PL emission of (a) pure **Ia**; (b) pure **IIa**; (c) pure synthesized CdS.

When we analyzed the photoluminescence of pure CdS, pure **Ia** and pure **IIa**, we observed that pure CdS has the lowest PL intensity of all the samples examined (see Insert in Figure 9). Pure **Ia** has a higher PL intensity than pure **IIa**, which could be attributed to their structures. Pure **Ia** has a methoxy group (-OCH_3_) at the *meta* position of the phenyl ring in the middle of our structure. A methoxy substituent is an electron-donating group that could neutralize the positive charge of the aromatic ring [21]. Chaieb and co-workers [31] observed that placing such donating groups either on both ends or in the middle of the molecule led to fluorescence emission in their materials. This observation could explain why **Ia** has a higher PL emission than **IIa**.

Figure 9 shows the room temperature PL spectra of the CdS:**Ia** nanocomposite at different mass compositions (0.1:1.0 to 1.0:1.0 w/w of CdS:**Ia**). The spectrum of pure **Ia** showed an emission peak at 538 nm and a shoulder at 479 nm. In this study, we compared the intensity of the peak relative to the matrix (**Ia** or **IIa**) instead of to the pure CdS. Little PL emission was exhibited by the pure synthesized CdS nanoparticles using a similar method [20] when compared to the PL emission by **Ia** or **IIa**. When CdS was introduced into the nanocomposites (20% by weight), the intensity of the peak decreased to half. The maximum peak and the shoulder peaks, however, remained the same and a new shoulder appeared at 516 nm. When 30% by weight of CdS was added, the intensity of the peak at 516 nm became larger than that of the peaks at 479 nm and 538 nm. The spectra for the nanocomposite with a mass composition of 0.4:1.0 showed a similar pattern as the spectra for the nanocomposite with a mass composition of 0.3:1.0. The peaks for the nanocomposite with a mass composition of 0.6:1.0 remained the same, although the PL intensity decreased slightly. However, the intensities of the peaks decreased drastically to 25% of the intensity of the CdS:**Ia** nanocomposite with a mass composition of 0.2:1.0. The introduction of CdS into **Ia** resulted in strong quenching of the PL intensity of the matrix. This quenching effect intensified with increasing amounts of CdS in the nanocomposite. Lu and colleagues (2003)[32] reported a similar result when greater amounts of CdS were introduced (as a coating) into their ZnS micellar particle solutions. In the current study, the luminescence intensity of the matrix clearly decreased with increasing CdS content. This behavior could be explained by the disappearance of the luminescence centers, by the existence of non-radiative defects that were induced by CdS or by absorption of the PL by CdS. As demonstrated in the earlier study, the CdS particles mainly absorbed below 500 nm. In this case, the absorption of PL emission by CdS would result in a shift of the matrix PL to a higher wavelength, which was not observed. Further PL investigations are needed to understand the origin of the PL quenching that is induced by the presence of CdS.

**Figure 10:**
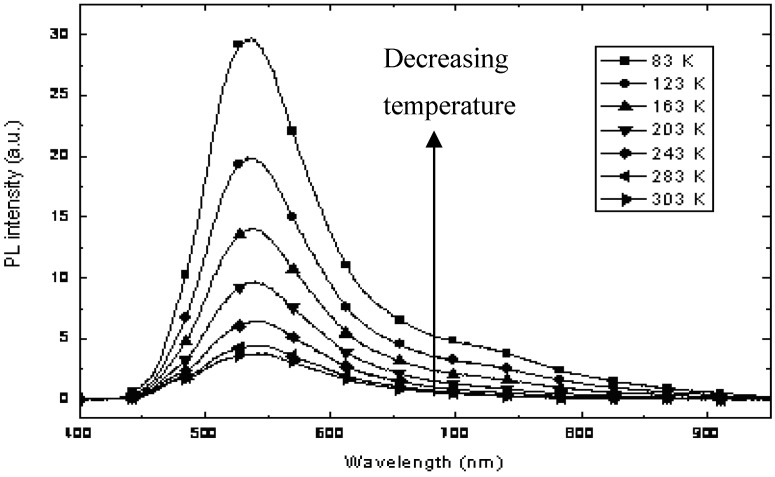
Temperature dependence of the PL of CdS: **Ia** nanocomposite at 0.2:1:0 (w/w).

The CdS:**Ia** nanocomposite with a mass composition of 0.2:1.0 was selected for further studies that were focused on the temperature dependence of photoluminescence in the range of 773–03 K, as depicted in Figure 10. As the temperature decreased, the peak intensity at 532 nm, which originated from **Ia**, increased gradually. Moreover, a peak around 750 nm increased continuously as the temperature decreased. This peak is attributed to the luminescence of CdS, as confirmed in our earlier PL study. The temperature dependence of the PL of the CdS:**Ia** nanocomposites clearly showed that non-radiative channels exist in the nanocomposite. The PL at 750 nm for CdS was attributed to defect states. The results obtained in this study suggest that the defect states (probably due to sulfur vacancies) were passivated by the matrix.

**Figure 11:**
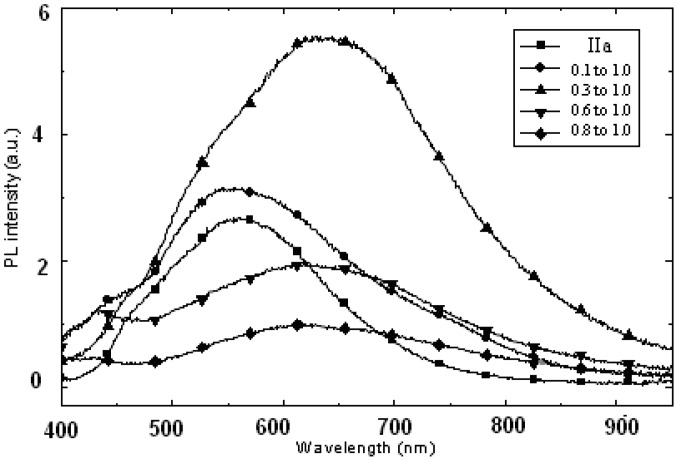
Photoluminescence study of CdS:**IIa** nanocomposites at different mass compositions (w/w).

Figure 11 shows the photoluminescence study at room temperature for the CdS:**IIa** nanocomposites at various mass compositions. Pure **IIa** has a PL peak at 559 nm and a shoulder at 460 nm. When the CdS nanocomposite had a mass composition of 0.1:1.0, the PL intensity of the matrix increased slightly and the peaks also became broader due to a shoulder at a high wavelength. Nonetheless, the PL intensity increased and red shifted to 640 nm when the CdS nanocomposite had a mass composition of 0.3:1.0. Conversely, the PL intensity decreased when more CdS was introduced, although the peak remained in the 6126–22 nm region. For higher CdS contents, the PL intensity the PL intensity decreased with the CdS content.

**Figure 12:**
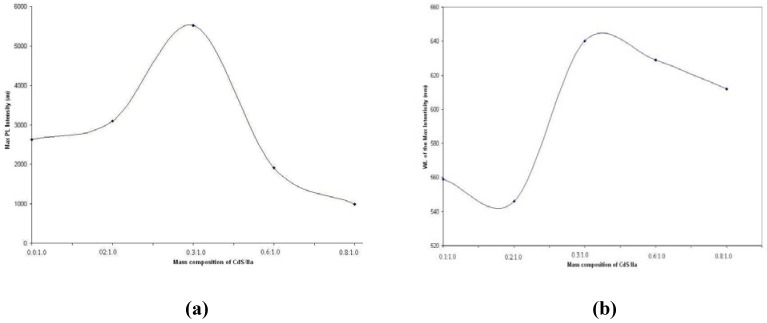
(a) The maximum intensities against various mass compositions; (b) The wavelength of the maximum intensity against various mass compositions for nanocomposites **IIa**.

If we plot the maximum PL intensities against various mass compositions for nanocomposite **IIa** (see Figure 12a), the results corresponded well with the findings from Ushakov and co-workers (2006) [33] in their work on CdS nanoparticles and polyethylene nanocomposites at different mass percentages (from 0% to 40%). These researchers obtained the photoluminescence spectrum at 20 mass % CdS nanoparticles and found that the CdS nanoparticles suppress the matrix radiation and enhanced the radiation intensity of the nanomaterial. The authors claimed that the results were associated with the high quantum efficiency of the photoluminescence, and this feature was due to a higher concentration of CdS nanoparticles with fewer imperfections. Thus, the radiation intensity of the nanomaterial was increased. The plot of the wavelength of the maximum intensity against various mass compositions is illustrated in Figure 12b.

**Figure 13:**
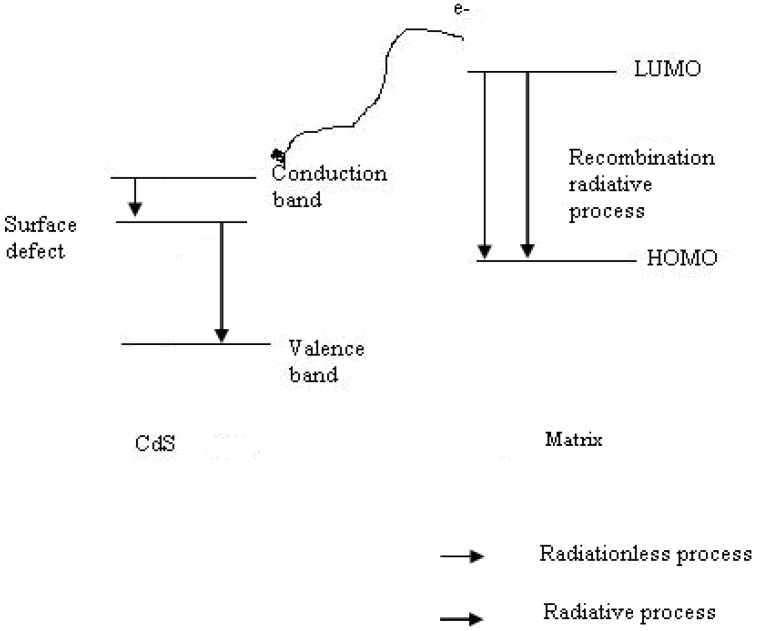
The proposed kinetic scheme in CdS nanocomposites.

In this study, two different PL contributions were observed. The PL bands around 550 nm and around 700 nm were attributed to the matrix and to the CdS nanoparticles, respectively. With an increasing quantity of CdS, the PL band shift toward higher wavelengths because of the increasing influence of the CdS nanoparticles. Moreover, the disappearance of the PL of the matrix for the high CdS concentration suggested that an energy transfer process from the matrix to CdS took place. The excitation of the mixture could lead to radiative transitions from the matrix or from CdS, or alternatively, could lead to radiative transfer from the matrix to CdS followed by the PL of CdS. These processes are illustrated in Figure 13.

**Figure 14:**
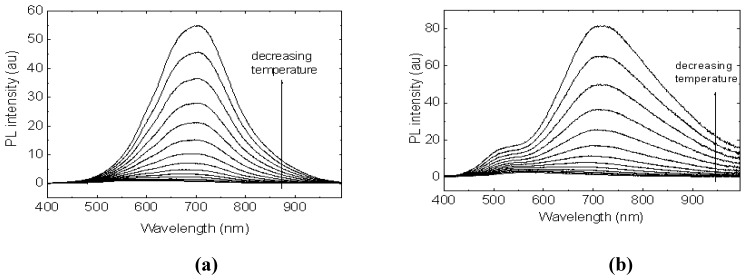
Temperature dependence studies on PL in CdS: **IIa** nanocomposites at (a) 0.4:1.0 (b) 0.2:1.0 (w/w).

The temperature dependence of photoluminescence was then studied on **IIa** nanocomposites of 0.4:1.0 and 0.2:1.0 as seen in Figure 14. The study was carried out in the range of 77 –300 K. The photoluminescence intensity increased gradually as the temperature decreased. In the case of CdS nanocomposite at 0.2:1.0, we could observe that the PL peak of CdS was increased as the temperature was gradually decreased, suggesting that thermally activated non-radiative processes exist in the matrix. Similarly, the PL peak for **Ia** at 540 nm was also intensified as the temperature was lowered. However, when more CdS were added (Figure 14a), the PL peak of CdS became dominant and no PL peak of the **Ia** was observed in the spectra. All of the peaks coalesced into a single peak at around 700 nm, and this peak represented the CdS peak in the mixtures. The introduction of CdS into **Ia** (ultimately reaching a mass composition of 0.3:1.0) enhanced the photoluminescence of the sample, and eventually the PL intensity dropped as more CdS was added. Nonetheless, the intensity peak for CdS in the CdS naocomposite was monitored to have increased when the temperature is decreased.

A comparison of the CdS nanocomposites of **Ia** and **IIa** in the temperature dependence studies showed that the introduction of CdS nanoparticles produced different results, depending on the sample. Addition of CdS into **Ia** quenched the photoluminescence intensity of **Ia**. However, the introduction of CdS into **IIa** enhanced the photoluminescence of the sample to a mass composition of 0.3:1.0 and the PL intensity dropped as more CdS was added. The intensity of the peak for CdS in the CdS:**IIa** nanocomposite was found to increase when the temperature was decreased. The reverse trend was observed when the CdS:**Ia** nanocomposite was studied over a range of temperatures. In contrast to the CdS:**IIa** sample, the **Ia** peak (rather than the CdS peak) responded when the temperature was decreased, except when the temperature was 77 K.

## 3. Experimental Section

### 3.1. Measurements

The 1D-NMR (^1^H) spectra were obtained using a Bruker 400 MHz spectrometer. The samples were prepared at a concentration of 100 mg·mL^-1^ in CDCl_3_. Tetramethylsilane (TMS) was used as the internal reference. All synthesized nanocomposites were characterized by powder X-ray diffraction (XRD). The data were collected on a SIEMENS D5000 X-ray diffractometer with a monochromatic Cu-Kα (λ = 1.5405 Å) radiation filter in the 2θ range of 0–100°. The average particle size and the selected area electron diffraction (SAED) analysis were obtained with a Philips CM20 transmission electron microscope (TEM) operating at 200 kV. A few drops of dispersion were placed on a carbon-coated copper grid, and the solvent was evaporated off. A Leica Cambridge S360 energy dispersive X-ray (SEM-EDX) instrument was used to observe the morphology and the elemental composition of the product. The powders were mounted onto the metal holder using double-sided tape, and then the powders were coated with gold. The particle size of the sample was analyzed using AnalySIS Docu Image Analyzer Ver. 3.1. The FT-IR spectra were measured on a Perkin-Elmer 2000 FT-IR instrument.

The differential scanning calorimetry (DSC) measurements were carried out with a Perkin-Elmer DSC7 instrument at a heating rate of 5 ºC min^-1^ in nitrogen. The liquid crystalline mesophase was measured using a Nikon Eclipse E600 polarizing optical microscope (POM) that was equipped with a Linkam THMS 600 hot stage, a Linkam TP93 temperature controller and a Linkam LNP cooling system.

For the photoluminescence (PL) experiments, the excitation wavelengths (313 and 334 nm) were obtained from the UV lines of a 200 W mercury arc lamp source (Oriel). We used a dichroic mirror to avoid the emission in the visible range of the source (green and yellow lines) and a band-pass filter to select the 313 and 334 nm lines. In addition, another filter was placed in front of the detector to avoid the entrance of the excitation lines that were diffused by the sample. This filter cuts off the light at wavelengths lower than 400 nm. The optical emission was analyzed using a monochromator (Triax 190, Jobin Yvon) equipped with a grating (150 grooves/mm) and a CCD camera that was cooled to 140 K. The acquisition time was around 1 s. The response of the detection system was precisely calibrated with a tungsten wire calibration source. A cryostat was used to perform the measurements at 77 K. The chemical compositions of the samples were characterized by X-ray photoemission spectroscopy (XPS, Mg Kα: hυ = 1253.6 eV) from ESCALAB MK II VG. The spectrometer energy calibration was accomplished with the Au4f7/2 photoelectron level using a gold foil. Due to surface electrostatic charges, which result in retarding effects, the corresponding energy scales of the XPS spectra were normalized using the fixed energy position of the C1s photoelectron line of the carbon contamination. The real value of C1s is between 284 [34] and 285 eV [35]. Most authors use 285 eV as the reference for C1s. Therefore, the value of 284 eV from Tong *et al.* [34] is shifted by 1 eV to have the same C1s energy as other journal reports [35]. Hence, the level is higher in binding energy.

The optical properties of the nanocomposites (dispersed in ethanol solution) were measured using a UV-Vis spectrophotometer (Hitachi U-2000). The solution was placed in a 1-cm quartz cuvette, and the spectrum was measured within the range of 200–800 nm. Micro Raman Spectroscopy (Jobin Yvon Horiba UV-VIS NIR micro-Raman) was performed at room temperature using a non-polarized Ar^+^ laser (514.9 nm) in a nearly back-scattering geometry. Low power beams (typically 20 mW at the sample surface) were used to avoid heating and other damage to the samples during the analysis. The resolution of the spectrometer was around 2 cm^-1^.

### 3.2. Synthesis

#### 3.2.1. Preparation of bisphenol N,N’-bis(4-hydroxy-3methoxy)-benzylidene-o-tolidine (**I**) and bisphenol N,N’-bis(4-hydroxy)-benzylidene-o-tolidine (**II**).

The preparation of these bisphenols was reported in our previous publication [36].

#### 3.2.2. Synthesis of 3,3’-dimethoxy-4,4’-di(hydroxyhexoxy)-N-benzylidene-o-Tolidine (**Ia**) and 4,4’-di(6-hydroxyhexoxy)-N-benzylidene-o-Tolidine (**IIa**)

1-Chloro-6-hexanol (0.4 mol) and sodium carbonate (0.45 mol) were mixed in 400 mL of dimethylformamide (DMF). Then, a solution of 0.2 mol of either **I** or **II** in DMF (60 mL) was added to the 500 mL reaction flask. The mixture was then stirred and refluxed at 130 ºC for 12 hours. The product was precipitated by pouring the resulting solution into cold distilled water (2 L). The precipitate was filtered, was washed several times with diethyl ether and was dried in a vacuum oven at 90 ºC. The final purification was carried out by re-crystallization from DMF:1-butanol (1:1) to give either light green crystals **(Ia)** or brown crystals **(IIa)**.


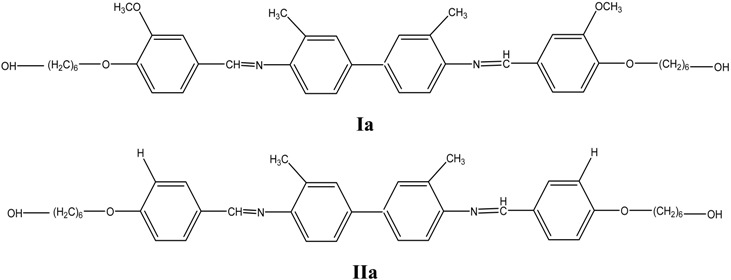


#### 3.2.3. Preparation of (1.0:1.0 w/w) CdS/**Ia** or **IIa** nanocomposite

Cadmium chloride (CdCl_2_·2.5H_2_O, 24 mg) and **Ia** or **IIa** (24 mg) were mixed in 15 mL of ethanol (Solution A). Solution B was prepared by dissolving 12 mg of sodium hydroxide (NaOH) in ethanol (12 mL). Solution B (10 mL) was added to solution A and the mixture was stirred. The mixture was refluxed at 160 ºC in an oil bath for 12 hours. A solution of thiourea ((NH_2_)_2_CS, 48 mg) in 15 mL of ethanol was added dropwise during the reaction. The obtained nanocomposite was collected by centrifugation and was washed repeatedly with ethanol before drying in a vacuum oven at 50 ºC for 24 hours. A series of different CdS:**Ia** and CdS:**IIa** mass compositions were also prepared.

## 4. Conclusions

CdS:**Ia** and CdS:**IIa** nanocomposites were successfully synthesized using a simple *in situ* technique. In this study, the PL emissions for CdS:**Ia** and CdS:**IIa** nanocomposites were in the red-shift region, suggesting that deep trap defects occurred in both cases. These red-shifted emissions could be the result of particles being in contact with one another. However, the PL analyses showed unique findings in both cases. The PL for the CdS:**Ia** nanocomposite, irrespective of the CdS concentration, exhibited lower PL relative to **Ia**. In contrast, addition of CdS to the **IIa** nanocomposite (up to a mass composition of 0.3:1.0) increased the PL, but emission dropped when the mass composition was 0.3:1.0 and greater. At the end of the study, we concluded that the **IIa** monomer shows a greater potential when CdS is added, especially at the ratio of 0.3:1.0. However, the addition of CdS quenched the PL emission in **Ia** regardless of the mass composition. Hence, the **IIa** monomers will be selected for future investigations in which we plan to polymerize the monomers and then investigate the physical and chemical properties of the resulting polymer with the addition of CdS nanoparticles. This investigation is still in the preliminary stages; the synthesis is useful only on the laboratory scale and has not been tested on larger scales. Nonetheless, this nanocomposite has the great potential of being a light-harvesting device once the experimental conditions are optimized.
